# Influenza A(H1N1)pdm09 Virus but Not Respiratory Syncytial Virus Interferes with SARS-CoV-2 Replication during Sequential Infections in Human Nasal Epithelial Cells

**DOI:** 10.3390/v14020395

**Published:** 2022-02-15

**Authors:** Clément Fage, Mathilde Hénaut, Julie Carbonneau, Jocelyne Piret, Guy Boivin

**Affiliations:** Research Center of the CHU de Québec, Department of Microbiology-Immunology and Infectious Diseases, Faculty of Medicine, Laval University, Quebec City, QC G1V 4G2, Canada; fage.clement@gmail.com (C.F.); mathilde.henaut@crchudequebec.ulaval.ca (M.H.); julie.carbonneau@crchudequebec.ulaval.ca (J.C.); jocelyne.piret@crchudequebec.ulaval.ca (J.P.)

**Keywords:** viral interference, influenza virus, SARS-CoV-2, respiratory syncytial virus, human nasal epithelium, interferon

## Abstract

The types of interactions between severe acute respiratory syndrome coronavirus 2 (SARS-CoV-2) and other respiratory viruses are not well-characterized due to the low number of co-infection cases described since the onset of the pandemic. We have evaluated the interactions between SARS-CoV-2 (D614G mutant) and influenza A(H1N1)pdm09 or respiratory syncytial virus (RSV) in the nasal human airway epithelium (HAE) infected simultaneously or sequentially (24 h apart) with virus combinations. The replication kinetics of each virus were determined by RT-qPCR at different post-infection times. Our results showed that during simultaneous infection, SARS-CoV-2 interferes with RSV-A2 but not with A(H1N1)pdm09 replication. The prior infection of nasal HAE with SARS-CoV-2 reduces the replication kinetics of both respiratory viruses. SARS-CoV-2 replication is decreased by a prior infection with A(H1N1)pdm09 but not with RSV-A2. The pretreatment of nasal HAE with BX795, a TANK-binding kinase 1 inhibitor, partially alleviates the reduced replication of SARS-CoV-2 or influenza A(H1N1)pdm09 during sequential infection with both virus combinations. Thus, a prior infection of nasal HAE with SARS-CoV-2 interferes with the replication kinetics of A(H1N1)pdm09 and RSV-A2, whereas only A(H1N1)pdm09 reduces the subsequent infection with SARS-CoV-2. The mechanism involved in the viral interference between SARS-CoV-2 and A(H1N1)pdm09 is mediated by the production of interferon.

## 1. Introduction

Severe acute respiratory syndrome coronavirus 2 (SARS-CoV-2) has rapidly emerged and spread throughout the world, causing a pandemic crisis with disastrous sanitary and economic consequences. So far, more than 5.7 million deaths have been reported globally (as of February 2022) and the economic losses associated with the pandemic have reached USD 16 trillion in the United States [1].

For decades, seasonal influenza viruses and respiratory syncytial virus (RSV) were the two major causes of respiratory tract infections [2,3]. However, since the emergence of SARS-CoV-2, epidemiologic studies have demonstrated that the number of influenza cases has decreased markedly in the Northern and Southern Hemispheres [4,5,6,7], whereas RSV activity occurred at an unusual time [8,9,10]. For the 2020–2021 season, only 69 influenza cases were reported in Canada, which was lower than the average of 52,169 influenza detections recorded during the past six seasons [11]. Regarding RSV, only 986 cases were detected in Canada for the 2020–2021 season, which was around 20 times lower than the 18,916 cases reported during the 2019–2020 season [12]. However, a large RSV epidemic occurred during the spring/summer of 2021 in North America [13]. So far, it is not clear which factors (biological, societal or both) are involved in the decreased number of influenza infections during the COVID-19 pandemic. Indeed, social distancing rules and several sanitary measures put in place to slow the transmission of SARS-CoV-2 also affected the spread of respiratory viruses such as the influenza virus and RSV [14]. Therefore, the impact of SARS-CoV-2 co-infections with influenza viruses or RSV on clinical outcome is still debated because only a low number of co-detections have been documented since the onset of the pandemic.

Currently, little is known about interactions between SARS-CoV-2 and other respiratory viruses after concurrent or sequential infection of the respiratory tract. Different viral interactions can be positive (additive or synergistic) or negative (antagonistic) depending on if the first virus enhances or reduces infection and the replication of the second virus. Positive interactions mainly result in co-infections, whereas negative interactions, also called viral interference, are mediated by the interferon (IFN) response induced by a first virus that prevents the infection and replication of a second virus [15]. For instance, the prior infection of a reconstituted human airway epithelium (HAE) with human rhinoviruses was shown to prevent the replication of influenza virus [16] and SARS-CoV-2 [17,18,19] by a mechanism involving the innate immune response. A recent epidemiological study reported that the likelihood of testing positive for SARS-CoV-2 was 58% lower among influenza-positive cases, with an odds ratio of 0.42; 95% confidence interval 0.31–0.56 [20]. Therefore, we suggest that viral interference may occur between SARS-CoV-2 and other respiratory viruses such as influenza and RSV, which could partially contribute to the small number of clinical co-infection cases reported to date [7,21].

In this study, we sought to assess the interactions between SARS-CoV-2 and major respiratory viruses such as influenza A(H1N1)pdm09 and RSV-A2 in nasal HAE infected, simultaneously or sequentially, with pairs of viruses. Our results show that a prior infection with SARS-CoV-2 interferes with the replication kinetics of both viruses, whereas influenza A(H1N1)pdm09 but not RSV-A2 prevents the replication of a subsequent SARS-CoV-2 infection by a mechanism relying on IFN production.

## 2. Materials and Methods

### 2.1. Cells, Viruses and Biosafety

ST6GalI-MDCK (Madin–Darby canine kidney) cells (kindly provided by Dr Y. Kawaoka from the University of Wisconsin, Madison, WI, USA), Vero E6 (green monkey kidney) cells (American Type Culture Collection (ATCC) no. CRL-1586; Manassas, VA, USA) and Hep-2 (human epithelial carcinoma) cells (ATCC no. CCL-23) were cultured in minimum essential medium (MEM; Invitrogen, Carlsbad, CA, USA) supplemented with 10% fetal bovine serum (FBS; Invitrogen) and 1% HEPES. Nasal HAE (MucilAir™; pool of donors, EP02MP) and culture medium were provided by Epithelix Sàrl (Geneva, Switzerland). Epithelial cells obtained after nasal polyps removal surgery from 14 donors were cultured for 45 days to reconstitute fully differentiated nasal HAE composed of ciliated, goblet and basal cells [22]. Nasal HAE was cultured in 24-well inserts at the air-liquid interface at 37 °C with 5% CO_2_ following the manufacturer’s instructions. Except when otherwise stated, the medium at the basal pole of nasal HAE was replaced by fresh medium every 48 h during culture maintenance and experimental protocols.

Influenza A/California/7/2009(H1N1) virus (referred to as A(H1N1)pdm09) was amplified on ST6GalI-MDCK cells, and viral titers were quantified by the plaque assay. SARS-CoV-2 strain Quebec/CHUL/21697 was isolated from a clinical sample (nasopharyngeal swab) in March 2020 in Quebec City, Canada. SARS-CoV-2 RNA was sequenced by MinION technology (Oxford Nanopore technologies, Oxford, UK), and the D614G substitution was detected in the spike protein. The strain was passaged twice on Vero E6 cells and viral titers were quantified by the plaque assay. The RSV strain A2 (ATCC no. VR-1540) was amplified on Hep-2 cells. Viral titers were quantified on Hep-2 cells by immunostaining with a goat anti-RSV primary antibody (MD-05-0391; Cedarlane, Burlington, ON, Canada) and a horseradish peroxidase-labeled rabbit anti-goat IgG secondary antibody (HAF017; Cedarlane) as described in [23]. The True-Blue™ Peroxidase Substrate (KPL, Gaithersburg, MD, USA) was used to reveal infected foci.

All experimental work using infectious SARS-CoV-2 was performed in a Biosafety Level 3 (BSL3) facility at the CHU de Québec-Université Laval with appropriate air respirators and protective equipment.

### 2.2. Infections of Nasal HAE

Before each infection, apical poles of nasal HAE were gently washed once with 200 μL of pre-warm Opti-MEM (Gibco; ThermoFisher Scientific, Waltham, MA, USA). The multiplicity of infection (MOI) for all viruses was calculated based on manufacturer information, considering that each nasal HAE insert comprised 500,000 fully differentiated cells. In our experiments, we chose to infect nasal HAE with the different respiratory viruses at low MOIs (from 0.015 to 0.02 in single and dual infections) to prevent rapid destruction of the epithelium by the first virus, which could affect the course of the second infection. Tissue integrity was assessed by measurement of trans-epithelial electrical resistance (TEER) with a Millicell^®^ ERS-2 Voltohmmeter (Millipore-Sigma, St. Louis, MO, USA) during a single infection with each virus (data not shown).

**Figure 1:**
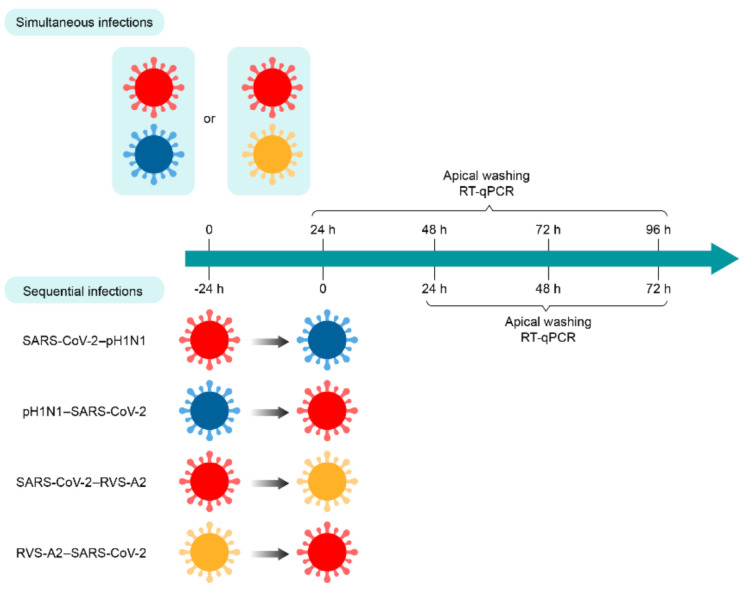
Timelines of simultaneous and sequential infections of nasal human airway epithelium (HAE) with SARS-CoV-2 and A(H1N1)pdm09 or RSV-A2 virus combinations. Nasal HAE was simultaneously or sequentially (24 h apart) infected with SARS-CoV-2/influenza A(H1N1)pdm09 or SARS-CoV-2/RSV-A2 virus combinations at the apical pole. Matched single virus infection conditions were performed in parallel. Viral loads of SARS-CoV-2, A(H1N1)pdm09 and RSV-A2 were determined by RT-qPCR in apical pole washings harvested every 24 h. Key: SARS-CoV-2, influenza A(H1N1)pdm09 virus (pH1N1) and RSV-A2 are represented in red, blue and yellow, respectively.

For simultaneous infection with SARS-CoV-2 and influenza viruses, the apical poles of nasal HAE were first infected with the A(H1N1)pdm09 virus in 200 µL of Opti-MEM for 30 min at 37 °C under a 5% CO_2_ atmosphere. Then, the inoculum was removed, and nasal HAE was quickly incubated with SARS-CoV-2 in 200 µL of Opti-MEM for 30 min at 37 °C with 5% CO_2_. Nasal HAE was infected with either SARS-CoV-2 or influenza A(H1N1)pdm09 virus in parallel to monitor single-infection kinetics. The MOI was set at 0.015 in single and dual infections. After the adsorption period, the inoculum was removed, and nasal HAE was cultured at the air–liquid interface. For sequential infection with both viruses, the same protocol was followed except that a delay of 24 h was set between the first and second infections (Figure 1).

For simultaneous infection with SARS-CoV-2 and RSV-A2, the apical poles of nasal HAE were infected with either SARS-CoV-2, RSV-A2 or a mixture containing both viruses in 300 µL of Opti-MEM for 1 h at 37 °C with 5% CO_2_. The MOI was set at 0.02 in single and dual infections. After this adsorption period, the inoculum was removed, and nasal HAE was cultured at the air–liquid interface. For sequential infections, nasal HAE was inoculated with the primary and secondary viruses 24 h apart or with each single virus following a similar procedure (Figure 1).

For all experimental conditions, viral production was evaluated from apical washes with 200 μL of pre-warm Opti-MEM for 10 min at 37 °C, under 5% CO_2_ at the post-infection (p.i.) times indicated in Figure 1.

### 2.3. Pretreatment with BX795

BX795 (Millipore-Sigma) was reconstituted in dimethyl sulfoxide (at a concentration of 10 mM) and used to treat the basal pole of nasal HAE, as previously described [17]. Briefly, 24 h before infection, nasal HAE growth medium was supplemented with 6 μM of BX795. The medium at the basal pole of HAE was replaced by 600 µL of a fresh medium containing BX795, and this step was repeated every 24 h during the infection to maintain treatment efficacy. Viral production was evaluated from apical washes at different p.i. times as described above.

### 2.4. Viral Load by RT-qPCR

A volume of 100 μL of apical washes was used to extract viral RNA (MagNA Pure LC, Total nucleic acid isolation kit, Roche Molecular System, Laval, QC, Canada). The viral RNA loads were determined by one-step reverse-transcription quantitative PCR (RT-qPCR) assays by using primers and probes to target the E gene of SARS-CoV-2 [24], the M gene of influenza A/H1N1 (available upon request) and the N gene of RSV-A2 [25] with the QuantiTect Virus + ROX Vial Kit (Qiagen, Toronto, ON, Canada) on a LightCycler^®^ 480 system (Roche Molecular System).

### 2.5. Statistical Analyses

All experiments were performed twice with duplicate nasal HAE, and results were expressed as mean ± SEM. Statistical analyses were performed using GraphPad Prism 5 software (GraphPad Software, San Diego, CA, USA) with a Mann–Whitney statistical test. A *p* value ≤ 0.05 was considered statistically significant.

## 3. Results

### 3.1. SARS-CoV-2 Interferes with RSV-A2, but Not with Influenza A(H1N1)pdm09 Replication during Simultaneous Infections

**Figure 2:**
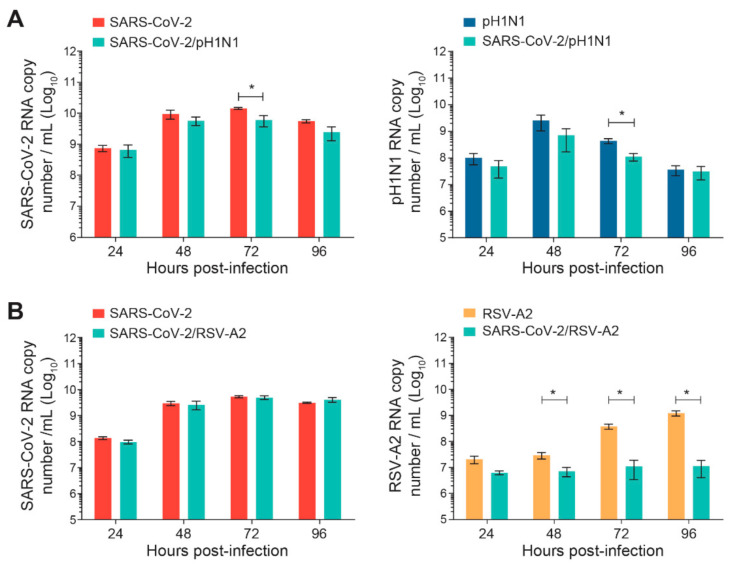
Replication kinetics of SARS-CoV-2 and influenza A(H1N1)pdm09 or RSV-A2 on nasal human airway epithelium (HAE) during simultaneous infections. Nasal HAE was simultaneously infected with SARS-CoV-2 and influenza A(H1N1)pdm09 (**A**) or SARS-CoV-2 and RSV-A2 (**B**) at the apical pole. Single infections with each virus were performed in parallel. The MOIs were set at 0.015 (SARS-CoV-2/A(H1N1)pdm09 series) or 0.02 (SARS-CoV-2/RSV-A2 series) in single and dual infections. The viral loads of SARS-CoV-2, influenza A(H1N1)pdm09 and RSV-A2 were determined by RT-qPCR. Results represent the mean ± SEM of 2 independent experiments with duplicate HAE. * *p* < 0.05 by the Mann–Whitney test. pH1N1, influenza A(H1N1)pdm09 virus.

The nasal HAE was infected simultaneously with SARS-CoV-2 and influenza A(H1N1)pdm09 or SARS-CoV-2 and RSV-A2 pairs of viruses, as shown in Figure 1. The replication kinetics of each virus were compared to those of single-infection conditions for 96 h p.i. No significant differences were observed during SARS-CoV-2 and A(H1N1)pdm09 simultaneous infections, except at 72 h p.i. for both viruses (*, *p* < 0.05; Figure 2A). However, during simultaneous infections with SARS-CoV-2 and RSV-A2, the replication kinetics of RSV-A2significantly decreased at 48, 72 and 96 h p.i. (*, *p* < 0.05; Figure 2B) but no effect was seen on the SARS-CoV-2 viral load.

### 3.2. Viral Interference between SARS-CoV-2 and Other Respiratory Viruses Is Observed during Sequential Infections

**Figure 3:**
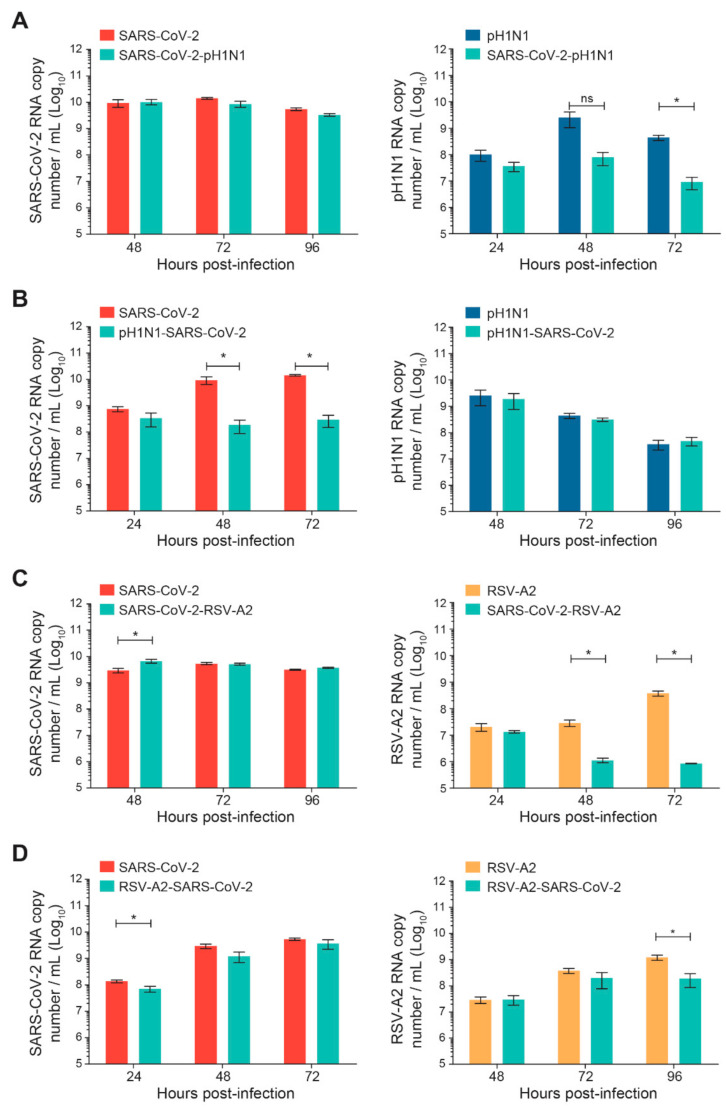
Replication kinetics of SARS-CoV-2 and influenza A(H1N1)pdm09 or RSV-A2 virus combinations on nasal human airway epithelium (HAE) during sequential infections compared to matched single-infection conditions. Nasal HAE was sequentially infected with SARS-CoV-2/influenza A(H1N1)pdm09 (**A**,**B**) or SARS-CoV-2/RSV-A2 (**C**,**D**) virus combinations 24 h apart at the apical pole. Single infections with each virus were performed in parallel. The MOIs were set at 0.015 (SARS-CoV-2/A(H1N1)pdm09 series) or 0.02 (SARS-CoV-2/RSV-A2 series) in single and dual infections. Viral loads of SARS-CoV-2, influenza A(H1N1)pdm09 and RSV-A2 were determined by RT-qPCR. Results represent the mean ± SEM of 2 independent experiments with duplicate HAE. ns, not significant; * *p* < 0.05 by the Mann–Whitney test. pH1N1, influenza A(H1N1)pdm09 virus.

Sequential infections of nasal HAE with SARS-CoV-2 and influenza A(H1N1)pdm09 or RSV-A2 were performed with a time interval of 24 h, as shown in Figure 1. The replication kinetics were determined for the primary and secondary viruses and compared to their matched single-infection conditions. When SARS-CoV-2 infection was followed by influenza A(H1N1)pdm09, no significant difference was observed in SARS-CoV-2 replication (Figure 3A). However, the replication kinetics of influenza A(H1N1)pdm09 were impacted by a prior SARS-CoV-2 infection. Indeed, the viral load of influenza A(H1N1)pdm09 was reduced by 2 log_10_ at 72 h p.i. (* *p* < 0.05; Figure 3A). In the nasal HAE infected with influenza virus followed by SARS-CoV-2, SARS-CoV-2 viral load was decreased by approximately 2 log_10_ at 48 and 72 h p.i. (* *p* < 0.05; Figure 3B) whereas the A(H1N1)pdm09 virus replication was not significantly affected (Figure 3B).

When SARS-CoV-2 infection was followed by RSV-A2, the replication of SARS-CoV-2 was increased at 48 h p.i. (* *p* < 0.05; Figure 3C), whereas the viral load of RSV-A2 was reduced by more than 1 log_10_ at 48 and 72 h p.i. (* *p* < 0.05; Figure 3C). Finally, during sequential infections with RSV-A2 followed by SARS-CoV-2, the replication of SARS-CoV-2 was slightly reduced at 24 h p.i. (* *p* < 0.05; Figure 3D) but not at 48 and 72 h p.i. The viral load of RSV-A2 was significantly decreased by less than 1 log_10_ at 96 h p.i. (* *p* < 0.05; Figure 3D).

### 3.3. Inhibition of Type I IFN Response Induced by the First Virus Partially Restores Replication of the Second Virus in Sequential Infections with Influenza and SARS-CoV-2

Innate immune responses, such as type I IFN (IFN-I) production, triggered by a primary virus could mediate the interference observed in the secondary infection with an unrelated virus [15]. To test this hypothesis, the nasal HAE was pre-treated with 6 µM of BX795 compound, an inhibitor of TANK-binding kinase 1 [26], 24 h before the primary infection and treatment solutions were replaced every 24 h.

**Figure 4:**
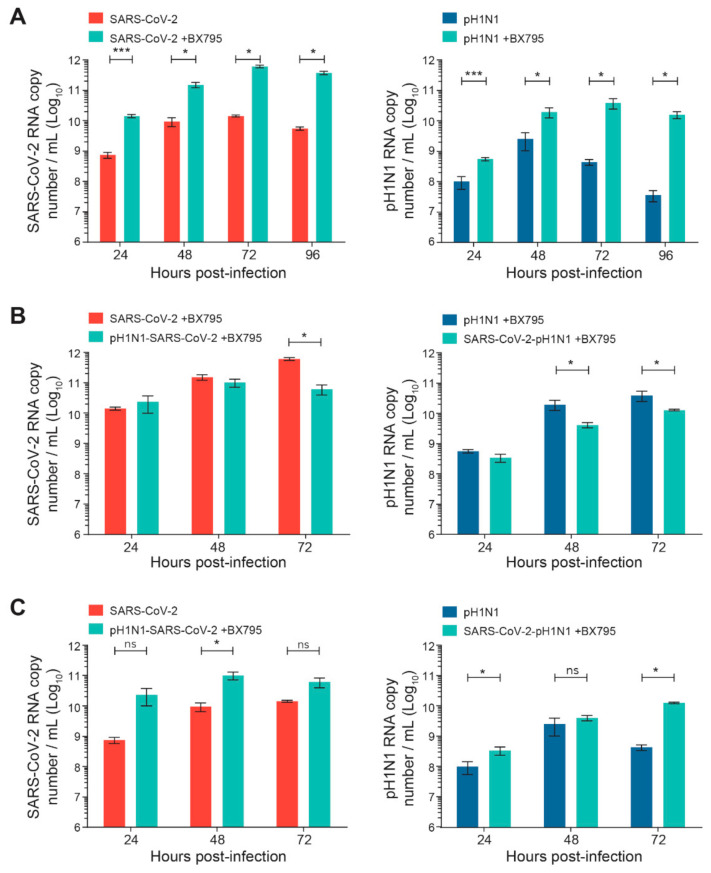
Effects of a pretreatment of nasal human airway epithelium (HAE) with BX795 on single or sequential infection with SARS-CoV-2 and influenza A(H1N1)pdm09 virus. Nasal HAE were pretreated with BX795 at the basal pole, and the medium was replaced by a fresh medium containing BX795 every 24 h during the infection. After 24 h, HAE was sequentially infected (24 h apart) with SARS-CoV-2/influenza A(H1N1)pdm09 virus combinations at the apical pole. Single infections with each virus were performed in parallel. The MOI was set at 0.015 in single and dual infections. Viral loads of both viruses were determined by RT-qPCR. (**A**) Effects of BX795 on replication kinetics of SARS-CoV-2 or influenza A(H1N1)pdm09 virus during single infections. (**B**) Effects of BX795 on the replication kinetics of SARS-CoV-2 and influenza A(H1N1)pdm09 during sequential and single infections. (**C**) Comparison of the replication kinetics of SARS-CoV-2 and A(H1N1)pdm09 during sequential infections in presence of BX795 and single infections without BX795. Results are the mean ± SEM of 2 independent experiments performed in duplicate in HAE. ns, not significant; * *p* < 0.05; *** *p* < 0.01 by Mann–Whitney test. pH1N1, influenza A(H1N1)pdm09 virus.

The kinetics of the replication of SARS-CoV-2 or A(H1N1)pdm09 single infection were first evaluated in the nasal HAE, pretreated or not pretreated with BX795. As expected from published data [26], such pretreatment significantly increased the viral replication of SARS-CoV-2 or A(H1N1)pdm09 (by ≥ 1–2 Log_10_) compared to non-pretreated cells (* *p* < 0.05 and *** *p* < 0.001, respectively; Figure 4A).Interestingly, BX795 pretreatment before sequential infections with SARS-CoV-2 and A(H1N1)pdm09 partially restored the reduced replication of the secondary virus (Figure 4B). The pretreatment with BX795 significantly increased the viral loads of secondary SARS-CoV-2 or A(H1N1)pdm09 viruses compared to those of the corresponding single infections without BX795 pretreatment (* *p* < 0.05 for both; Figure 4C), indicating a partial restoration of replication for both viruses.

## 4. Discussion

In this study, we assessed the interactions between SARS-CoV-2 and two common respiratory viruses, influenza A(H1N1)pdm09 and RSV-A2, using an ex vivo model consisting of reconstituted human nasal epithelium. Nasal HAE cells are composed of fully differentiated human cell types such as ciliated, goblet and basal cells. This ex vivo model is more relevant than human-immortalized cell lines (e.g., A549 or Calu-3) or non-human cell lines (e.g., Vero E6) to study viral interactions, viral fitness or the effects of potential therapeutic agents [27]. Moreover, the study of viral interactions in human nasal epithelium is fundamental as the nasopharyngeal mucosa constitutes the first site of natural respiratory infections, and the viral interactions occurring therein could affect the potential dissemination of viruses towards the bronchial mucosa and the lungs. This model also has the advantage of allowing the study of viral interactions following simultaneous and sequential infections with a defined inoculum of specific strains of respiratory viruses. However, one of its potential limitations is the absence of immune cells such as interstitial and alveolar macrophages, as well as cells involved in adaptive immunity, which are essential for assessing virus neutralization and clearance.

Our results first demonstrate that no viral interactions between SARS-CoV-2 and the influenza A(H1N1)pdm09 virus were observed during simultaneous infection. When both infections were initiated 24 h apart, the replication kinetics of the secondary viruses, either A(H1N1)pdm09 or SARS-CoV-2, were significantly decreased by the first infection, whereas the viral load of the primary viruses was not affected at any time p.i. These results are partially in agreement with a recent study evaluating the interactions between SARS-CoV-2 and influenza A in the nasal HAE model [19]. Indeed, Essaidi-Laziosi et al. also reported that a primary infection with the influenza A(H1N1)pdm09 virus significantly reduced secondary SARS-CoV-2 replication [19]. However, a prior infection with SARS-CoV-2 did not affect the replication of influenza A, which is contrary to our observations. This discrepancy may be due to some divergences in the experimental protocols. Indeed, we used a similar MOI of 0.015 for both viruses, whereas Essaidi-Laziosi et al. used a 100-fold lower MOI for A(H1N1)pdm09 than for SARS-CoV-2 (0.001 vs. 0.1) [19]. Viral strains and experimental protocols for the infection of nasal HAE were also different in both studies. Moreover, the temperature of incubation was higher in our experiments (37 °C vs. 33 °C). The impact of influenza viruses on SARS-CoV-2 replication is still debated in the literature. In agreement with our observations and those of Essaisi-Laziosi et al. [19], Achdout et al. reported that the sequential infection of transgenic mice expressing the human angiotensin-converting enzyme 2 (ACE2) under a cytokeratin 18 promoter (K18-hACE2) with influenza A virus and SARS-CoV-2, 48 h apart, resulted in a significant decrease in SARS-CoV-2 viral loads in the lungs and nasal turbinates [28]. However, these authors did not assess the outcome of a sequential infection with SARS-CoV-2 prior to the influenza virus. Furthermore, two animal studies demonstrated that the sequential infection with A(H1N1)pdm09 one day prior to SARS-CoV-2 resulted in lower SARS-CoV-2 viral loads in the lungs of hamsters and ferrets compared to a single SARS-CoV-2 infection [29,30]. Nonetheless, Zhang et al. also showed that a sequential infection of hamsters with SARS-CoV-2 followed by A(H1N1)pdm09 increased the viral load of SARS-CoV-2 in lung tissues compared to a single infection [29]. Bai et al. also demonstrated that a pre-infection with influenza A virus promoted the infectivity of SARS-CoV-2 in several cultured cell lines (e.g., Vero E6, A549 and Calu-3) as well as in transgenic K18-hACE2 mice infected with both viruses at a 12 h interval [31]. The diversity of the conclusions from the interactions between SARS-CoV-2 and influenza A virus may be related to differences in the models, viral strains or experimental protocols used in these studies. For instance, this discrepancy could be explained by different time intervals between sequential infections. Indeed, the sequence and timing of infections could have an important impact on viral interference. Studies on ferrets showed that the time interval between sequential infections with different viruses (i.e., influenza and RSV) was a determinant for the occurrence of a viral interference or co-infection and was related to the production of immune mediators induced by the first virus [32,33]. Viral interference occurred during the period of production of immune mediators, whereas co-infection was observed before and after this window period. In our experiments, the pretreatment of nasal HAE with BX795 before sequential infection with SARS-CoV-2/A(H1N1)pdm09 virus combinations partially restored the replication kinetics of the secondary virus. This suggests that viral interference between these pairs of viruses could be mediated by the production of IFN induced by the primary virus. In this respect, Rand et al. reported that the replication of SARS-CoV-2 in Calu-3 cells was inhibited by a treatment based on influenza-A-defective particles through the production of IFN-I and IFN-III [34].

Our results also showed that SARS-CoV-2 was able to decrease RSV-A2 viral load independently of the mode of infection (simultaneous or sequential). However, RSV-A2 did not interfere with SARS-CoV-2 replication in any conditions. In the ferret model, A(H1N1)pdm09 also prevented a subsequent infection with RSV, whereas RSV reduced only the morbidity associated with influenza but not the infection [33]. Interestingly, a previous study analyzing RSV and human metapneumovirus (hMPV) interactions demonstrated that RSV was able to decrease hMPV viral loads during simultaneous and sequential infections in nasal HAE by a mechanism mediated by the IFN response induced by the first virus [35]. Moreover, this study also showed that hMPV was not able to interfere with RSV in any conditions. Another study also reported that RSV interferes with rhinovirus replication in nasal HAE infected, simultaneously or sequentially, with the two viruses through a mechanism involving IFN-I and IFN-III production [36]. The type of virus–virus interactions may be influenced by the mechanisms developed by respiratory viruses to escape the host immune response. In this respect, SARS-CoV-2 and the influenza virus have developed a broader range of multifaceted strategies to counteract IFN induction and signaling compared to RSV, hMPV and rhinovirus [37,38,39,40]. Therefore, we suggest that SARS-CoV-2 and the influenza virus could be less sensitive than RSV to the effects mediated by IFN. Importantly, the replication kinetics of influenza A(H1N1)pdm09 virus were more affected than that of SARS-CoV-2 in nasal HAE pretreated with recombinant human IFN-α2a (100 U/mL) at the basal pole (Figure S1). This corroborates the data showing that the pretreatment of nasal HAE with BX795 increased the replication kinetics of A(H1N1)pdm09 more effectively than that of SARS-CoV-2. Furthermore, the ability of the first virus to induce a potent IFN response is also a parameter that could predict the type of virus–virus interactions. In this respect, the expression of IFN-α and IFN-β was higher in lung tissues of ferrets infected with influenza A(H1N1)pdm09 virus compared to RSV on day 2 p.i. [33]. Finally, a mathematical model showed that the growth rate of RSV was slower than that of influenza A [41], which could partially explain why RSV-A2 had no effect on the replication kinetics of SARS-CoV-2. Additional studies with different strains and other models are needed to confirm this mechanism.

## 5. Conclusions

We report on the interactions between SARS-CoV-2 and the influenza A(H1N1)pdm09 or RSV-A2 virus combinations on nasal HAE. These observations could have implications for the prediction of epidemic peaks and pandemic waves and guide the recommendations made by public health authorities. A better understanding of the mechanisms involved in viral interference may help the development of novel modalities against respiratory viral infections based on this concept.

## Data Availability

The data presented in this study are available on request from the corresponding author.

## Supplementary Materials

The following supporting information can be downloaded at: https://www.mdpi.com/article/10.3390/v14020395/s1, Figure S1: Effects of a pretreatment of nasal human airway epithelium (HAE) with IFN-α2a on single infection with SARS-CoV-2 (**A**) and influenza A(H1N1)pdm09 virus (**B**). Nasal HAE was pretreated or not pretreated with recombinant human IFN-α2a (100 U/mL; Millipore-Sigma, St. Louis, MO, USA) at the basal pole and the medium was replaced by a fresh medium containing IFN-α2a every 24 h during the infection period. After 24 h, HAE was infected with SARS-CoV-2 or influenza A(H1N1)pdm09 (at a MOI of 0.015) at the apical pole. Viral RNA loads of both viruses were determined by RT-qPCR for 96 h post-infection. Results were from one independent experiment in duplicate HAE. Abbreviation: pH1N1, influenza A(H1N1)pdm09 virus.

## References

[1] Cutler D.M., Summers L.H. (2020). The COVID-19 Pandemic and the $16 Trillion Virus. JAMA.

[2] Brugger J., Althaus C.L. (2020). Transmission of and susceptibility to seasonal influenza in Switzerland from 2003 to 2015. Epidemics.

[3] Nam H.H., Ison M.G. (2019). Respiratory syncytial virus infection in adults. BMJ.

[4] Olsen S.J., Azziz-Baumgartner E., Budd A.P., Brammer L., Sullivan S., Pineda R.F., Cohen C., Fry A.M. (2020). Decreased Influenza Activity During the COVID-19 Pandemic—United States, Australia, Chile, and South Africa, 2020. MMWR Morb. Mortal. Wkly. Rep..

[5] Kim D., Quinn J., Pinsky B., Shah N.H., Brown I. (2020). Rates of Co-infection Between SARS-CoV-2 and Other Respiratory Pathogens. JAMA.

[6] Eisen A.K.A., Gularte J.S., Demoliner M., de Abreu Goes Pereira V.M., Heldt F.H., Filippi M., de Almeida P.R., Hansen A.W., Fleck J.D., Spilki F.R. (2021). Low circulation of Influenza A and coinfection with SARS-CoV-2 among other respiratory viruses during the COVID-19 pandemic in a region of southern Brazil. J. Med. Virol..

[7] Groves H.E., Piche-Renaud P.P., Peci A., Farrar D.S., Buckrell S., Bancej C., Sevenhuysen C., Campigotto A., Gubbay J.B., Morris S.K. (2021). The impact of the COVID-19 pandemic on influenza, respiratory syncytial virus, and other seasonal respiratory virus circulation in Canada: A population-based study. Lancet Reg. Health Am..

[8] Casalegno J.S., Ploin D., Cantais A., Masson E., Bard E., Valette M., Fanget R., Targe S.C., Myar-Dury A.F., Doret-Dion M. (2021). Characteristics of the delayed respiratory syncytial virus epidemic, 2020/2021, Rhone Loire, France. Eurosurveillance.

[9] Rybak A., Levy C., Jung C., Bechet S., Batard C., Hassid F., Zouari M., Cahn-Sellem F., Bangert M., Cohen R. (2021). Delayed Bronchiolitis Epidemic in French Primary Care Setting Driven by Respiratory Syncytial Virus: Preliminary Data from the Oursyn Study, March 2021. Pediatric Infect. Dis. J..

[10] Weinberger Opek M., Yeshayahu Y., Glatman-Freedman A., Kaufman Z., Sorek N., Brosh-Nissimov T. (2021). Delayed respiratory syncytial virus epidemic in children after relaxation of COVID-19 physical distancing measures, Ashdod, Israel, 2021. Eurosurveillance.

[11] Nwosu A., Lee L., Schmidt K., Buckrell S., Sevenhuysen C., Bancej C. (2021). National Influenza Annual Report, Canada, 2020–2021, in the global context. Can. Commun. Dis. Rep..

[12] Public Health Agency of Canada. Respiratory Virus Report, Week 34—Ending 28 August 2021. https://www.canada.ca/en/public-health/services/surveillance/respiratory-virus-detections-canada/2021-2022/week-34-ending-august-28-2021.html.

[13] Agha R., Avner J.R. (2021). Delayed Seasonal RSV Surge Observed During the COVID-19 Pandemic. Pediatrics.

[14] Wu D., Lu J., Liu Y., Zhang Z., Luo L. (2020). Positive effects of COVID-19 control measures on influenza prevention. Int. J. Infect. Dis..

[15] Piret J., Boivin G. (2022). Viral Interference between Respiratory Viruses. Emerg. Infect. Dis..

[16] Wu A., Mihaylova V.T., Landry M.L., Foxman E.F. (2020). Interference between rhinovirus and influenza A virus: A clinical data analysis and experimental infection study. Lancet Microbe.

[17] Dee K., Goldfarb D.M., Haney J., Amat J.A.R., Herder V., Stewart M., Szemiel A.M., Baguelin M., Murcia P.R. (2021). Human rhinovirus infection blocks SARS-CoV-2 replication within the respiratory epithelium: Implications for COVID-19 epidemiology. J. Infect. Dis..

[18] Cheemarla N.R., Watkins T.A., Mihaylova V.T., Wang B., Zhao D., Wang G., Landry M.L., Foxman E.F. (2021). Dynamic innate immune response determines susceptibility to SARS-CoV-2 infection and early replication kinetics. J. Exp. Med..

[19] Essaidi-Laziosi M.P., Alvarez C.M., Puhach O.P., Sattonnet-Roche P., Torriani G.P., Tapparel C.P., Kaiser L.M., Eckerle I.M. (2021). Sequential infections with rhinovirus and influenza modulate the replicative capacity of SARS-CoV-2 in the upper respiratory tract. Emerg. Microbes Infect..

[20] Stowe J., Tessier E., Zhao H., Guy R., Muller-Pebody B., Zambon M., Andrews N., Ramsay M., Lopez Bernal J. (2021). Interactions between SARS-CoV-2 and influenza, and the impact of coinfection on disease severity: A test-negative design. Int. J. Epidemiol..

[21] Nowak M.D., Sordillo E.M., Gitman M.R., Paniz Mondolfi A.E. (2020). Coinfection in SARS-CoV-2 infected patients: Where are influenza virus and rhinovirus/enterovirus?. J. Med. Virol..

[22] Tapparel C., Sobo K., Constant S., Huang S., Van Belle S., Kaiser L. (2013). Growth and characterization of different human rhinovirus C types in three-dimensional human airway epithelia reconstituted in vitro. Virology.

[23] Deffrasnes C., Hamelin M.E., Prince G.A., Boivin G. (2008). Identification and evaluation of a highly effective fusion inhibitor for human metapneumovirus. Antimicrob. Agents Chemother..

[24] Corman V.M., Landt O., Kaiser M., Molenkamp R., Meijer A., Chu D.K., Bleicker T., Brunink S., Schneider J., Schmidt M.L. (2020). Detection of 2019 novel coronavirus (2019-nCoV) by real-time RT-PCR. Eurosurveillance.

[25] Raymond F., Carbonneau J., Boucher N., Robitaille L., Boisvert S., Wu W.K., De Serres G., Boivin G., Corbeil J. (2009). Comparison of automated microarray detection with real-time PCR assays for detection of respiratory viruses in specimens obtained from children. J. Clin. Microbiol..

[26] Stewart C.E., Randall R.E., Adamson C.S. (2014). Inhibitors of the interferon response enhance virus replication in vitro. PLoS ONE.

[27] Pizzorno A., Padey B., Julien T., Trouillet-Assant S., Traversier A., Errazuriz-Cerda E., Fouret J., Dubois J., Gaymard A., Lescure F.X. (2020). Characterization and Treatment of SARS-CoV-2 in Nasal and Bronchial Human Airway Epithelia. Cell Rep. Med..

[28] Achdout H., Vitner E.B., Politi B., Melamed S., Yahalom-Ronen Y., Tamir H., Erez N., Avraham R., Weiss S., Cherry L. (2021). Increased lethality in influenza and SARS-CoV-2 coinfection is prevented by influenza immunity but not SARS-CoV-2 immunity. Nat. Commun..

[29] Zhang A.J., Lee A.C., Chan J.F., Liu F., Li C., Chen Y., Chu H., Lau S.Y., Wang P., Chan C.C. (2021). Coinfection by Severe Acute Respiratory Syndrome Coronavirus 2 and Influenza A(H1N1)pdm09 Virus Enhances the Severity of Pneumonia in Golden Syrian Hamsters. Clin. Infect. Dis..

[30] Bao L., Deng W., Qi F., Lv Q., Song Z., Liu J., Gao H., Wei Q., Yu P., Xu Y. (2021). Sequential infection with H1N1 and SARS-CoV-2 aggravated COVID-19 pathogenesis in a mammalian model, and co-vaccination as an effective method of prevention of COVID-19 and influenza. Signal Transduct. Target. Ther..

[31] Bai L., Zhao Y., Dong J., Liang S., Guo M., Liu X., Wang X., Huang Z., Sun X., Zhang Z. (2021). Coinfection with influenza A virus enhances SARS-CoV-2 infectivity. Cell Res..

[32] Laurie K.L., Guarnaccia T.A., Carolan L.A., Yan A.W., Aban M., Petrie S., Cao P., Heffernan J.M., McVernon J., Mosse J. (2015). Interval Between Infections and Viral Hierarchy Are Determinants of Viral Interference Following Influenza Virus Infection in a Ferret Model. J. Infect. Dis..

[33] Chan K.F., Carolan L.A., Korenkov D., Druce J., McCaw J., Reading P.C., Barr I.G., Laurie K.L. (2018). Investigating Viral Interference Between Influenza A Virus and Human Respiratory Syncytial Virus in a Ferret Model of Infection. J. Infect. Dis..

[34] Rand U., Kupke S.Y., Shkarlet H., Hein M.D., Hirsch T., Marichal-Gallardo P., Cicin-Sain L., Reichl U., Bruder D. (2021). Antiviral Activity of Influenza A Virus Defective Interfering Particles against SARS-CoV-2 Replication In Vitro through Stimulation of Innate Immunity. Cells.

[35] Geiser J., Boivin G., Huang S., Constant S., Kaiser L., Tapparel C., Essaidi-Laziosi M. (2021). RSV and HMPV Infections in 3D Tissue Cultures: Mechanisms Involved in Virus-Host and Virus-Virus Interactions. Viruses.

[36] Essaidi-Laziosi M., Geiser J., Huang S., Constant S., Kaiser L., Tapparel C. (2020). Interferon-Dependent and Respiratory Virus-Specific Interference in Dual Infections of Airway Epithelia. Sci. Rep..

[37] Li C., Wang T., Zhang Y., Wei F. (2020). Evasion mechanisms of the type I interferons responses by influenza A virus. Crit. Rev. Microbiol..

[38] Sa Ribero M., Jouvenet N., Dreux M., Nisole S. (2020). Interplay between SARS-CoV-2 and the type I interferon response. PLoS Pathog..

[39] Kikkert M. (2020). Innate Immune Evasion by Human Respiratory RNA Viruses. J. Innate Immun..

[40] Soto J.A., Galvez N.M.S., Benavente F.M., Pizarro-Ortega M.S., Lay M.K., Riedel C., Bueno S.M., Gonzalez P.A., Kalergis A.M. (2018). Human Metapneumovirus: Mechanisms and Molecular Targets Used by the Virus to Avoid the Immune System. Front. Immunol..

[41] Pinky L., Dobrovolny H.M. (2016). Coinfections of the Respiratory Tract: Viral Competition for Resources. PLoS ONE.

