# Supplementary material for: Influenza A(H1N1)pdm09 Virus but Not Respiratory Syncytial Virus Interferes with SARS-CoV-2 Replication during Sequential Infections in Human Nasal Epithelial Cells
Source: Viruses. 2022 Feb 15;14(2):395. doi: 10.3390/v14020395 (PMC8879759; doi:10.3390/v14020395)
Supplement: Supplementary file 1 [file viruses-14-00395-s001.zip › viruses-1594914-supplementary.pdf]

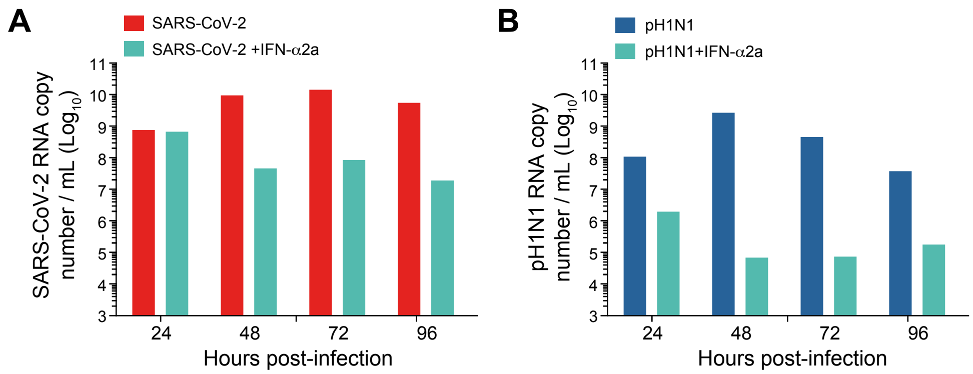

Figure S1. Effects of a pretreatment of nasal human airway epithelium (HAE) with IFN-α2a on single infection with SARS-CoV-2 (A) and influenza A(H1N1)pdm09 virus (B). Nasal HAE was pretreated or not pretreated with recombinant human IFN-α2a (100 U/mL; Millipore-Sigma, St. Louis, MO, USA) at the basal pole and the medium was replaced by a fresh medium containing IFN-α2a every 24 h during the infection period. After 24 h, HAE was infected with SARS-CoV-2 or influenza A(H1N1)pdm09 (at a MOI of 0.015) at the apical pole. Viral RNA loads of both viruses were determined by RT-qPCR for 96 h post-infection. Results were from one independent experiment in duplicate HAE. Abbreviation: pH1N1, influenza A(H1N1)pdm09 virus.
